# Endophytic *Streptomyces griseorubens* MEPSL1 from sweetpotato promotes plant growth and enhances γ-tocopherol accumulation

**DOI:** 10.1128/spectrum.03070-25

**Published:** 2026-02-17

**Authors:** Jingsheng Gu, Yiming Wang, Yu Sun, Yuxuan Xu, Yuanjiao Li, Chunyu Lin, Yue Ke, Lei Kai

**Affiliations:** 1 The Key Laboratory of Biotechnology for Medicinal and Edible Plants of Jiangsu Province, School of Life Sciences, Jiangsu Normal University 216827, Xuzhou, People's Republic of China; Instituto de Ecología, A.C. (INECOL), Pátzcuaro, Michoacán, Mexico

**Keywords:** *Streptomyces griseorubens*, sweetpotato, tocopherol biosynthesis, plant growth promotion

## Abstract

Endophytic actinomycetes are known to promote plant growth and improve crop nutritional quality through nutrient mobilization and secondary metabolite production. In this study, a multifunctional actinomycete strain was isolated from sweetpotato (*Ipomoea batatas* (L.) Lam.) and identified as *Streptomyces griseorubens* MEPSL1. Functional assays and genome analysis revealed that MEPSL1 exhibits several plant growth-promoting traits, including nitrogen fixation, phosphate solubilization, indole-3-acetic acid and siderophore production, 1-aminocyclopropane-1-carboxylate deaminase activity, and secretion of protease, amylase, and carboxymethyl cellulose-degrading activity. Inoculation of sweetpotato plants with MEPSL1 significantly increased the γ-tocopherol content in leaves, with HPLC analysis showing an increase of 38.31 μg g^−1^ dry weight compared to the control. Quantitative real-time PCR analysis confirmed the upregulation of four genes associated with tocopherol biosynthesis following treatment. Metabolite profiling also detected the presence of metabolites linked to tocopherol biosynthesis, suggesting a potential association in MEPSL1 cultures and a possible contribution to enhanced γ-tocopherol accumulation. These findings indicate that MEPSL1 can serve as an effective bioinoculant for improving both plant growth and the nutritional composition of sweetpotato leaves, with potential applications in sustainable agriculture and functional food development.

**IMPORTANCE:**

Streptomyces species are widely distributed in soils and plant tissues and are valued for their ecological safety and diverse biological functions. Here, we describe a multifunctional endophytic actinomycete, *Streptomyces griseorubens* MEPSL1, isolated from sweetpotato. This strain exhibits multiple growth-promoting traits and significantly increases γ-tocopherol accumulation in sweetpotato leaves, accompanied by the induction of key biosynthetic genes. Our findings highlight the role of endophytic actinomycetes in improving both crop performance and nutritional quality and point to their potential use in sustainable biofortification strategies.

## INTRODUCTION

Medicinal plants are broadly defined as species capable of preventing or alleviating disease (1). They represent an important reservoir of bioactive compounds and remain indispensable for both traditional and modern therapeutic applications (2). Beyond their role in drug manufacturing, medicinal plants are still widely used in traditional practices such as Chinese and Indian medicine, where they continue to serve as a primary source of treatment in many regions (3–5). The medical and economic value of these plants is therefore considerable, and with advances in natural product chemistry, an increasing number of modern drugs are being derived from plant metabolites (2).

Sweetpotato (*Ipomoea batatas* (L.) Lam.) is not only a major food crop but also recognized for its medicinal properties (6). It is nutritionally rich, supplying over 90% of essential nutrients per calorie except protein and niacin (7, 8). The storage roots provide abundant carbohydrates, vitamins, and minerals, while the leaves contain diverse secondary metabolites, including polyphenols, anthocyanins, flavonoids, and caffeic acid derivatives, which contribute antioxidant capacity and may reduce oxidative stress. Increasing attention has also been given to the therapeutic effects of sweetpotato extracts, which have been associated with anti-cancer, anti-diabetic, and anti-inflammatory activities (9).

Among its vitamins, vitamin E stands out for both human health and plant physiology. In sweetpotato, α-tocopherol and γ-tocopherol are the predominant isoforms (10). γ-Tocopherol, in particular, has drawn interest because of its strong antioxidant and anti-inflammatory properties, as well as anti-cancer potential (11). Unlike α-tocopherol, γ-tocopherol efficiently scavenges both reactive oxygen species and reactive nitrogen species, providing broader protection against oxidative damage (12). It also interferes with pro-inflammatory pathways, such as COX-2 and TNF-α signaling, thereby reducing chronic inflammation (13). Several studies have reported its ability to inhibit tumor proliferation, induce apoptosis, and suppress angiogenesis, especially in prostate and colon cancers (14). These findings suggest that γ-tocopherol may serve as a valuable natural compound for combating oxidative stress-related disorders.

To meet increasing demand for medicinal plants, chemical fertilizers are often used to increase yield, but this practice is associated with soil degradation, loss of fertility, and chemical residues in food products. Alternative approaches that maintain productivity while minimizing ecological impact are therefore needed. Beneficial microorganisms have emerged as promising candidates. These microbes can promote growth, enhance stress tolerance, and reduce dependency on chemical inputs by secreting metabolites, phytohormones, and protective enzymes. Endophytes, including bacteria, fungi, and actinomycetes, are particularly valued for their diversity and multifunctionality (15).

Actinomycetes, especially *Streptomyces* species, are among the dominant endophytes found in medicinal plants (16, 17). They are well known for producing secondary metabolites with pharmaceutical relevance, but they also support plant growth by solubilizing phosphate, fixing nitrogen, and producing phytohormones such as indole-3-acetic acid (IAA). In addition, they protect against pathogens through enzymes and metabolites, including cellulases, proteases, siderophores, and chitinases (18, 19). Some strains help plants cope with abiotic stress by lowering ethylene levels through the activity of 1-aminocyclopropane-1-carboxylate deaminase (ACCD), which breaks down the ethylene precursor ACC (1-aminocyclopropane-1-carboxylate) (20). Endophytes are also a rich source of bioactive natural products such as alkaloids, phenolics, saponins, quinones, flavonoids, and terpenoids (21). These functions indicate that endophytic actinomycetes may influence not only primary growth but also the accumulation of secondary metabolites with medicinal value.

In this study, we report the isolation of a multifunctional endophytic actinomycete, *Streptomyces griseorubens* MEPSL1, from sweetpotato. Through a combination of genomic, metabolomic, and molecular approaches, we show that MEPSL1 possesses diverse growth-promoting traits, including nutrient solubilization, phytohormone synthesis, and enzyme production. More importantly, inoculation with MEPSL1 significantly increased γ-tocopherol accumulation in sweetpotato leaves, accompanied by the upregulation of key biosynthetic genes. These results provide new insights into how endophytic actinomycetes may enhance both plant performance and the medicinal value of sweetpotato, offering a potential microbial strategy for sustainable biofortification.

## MATERIALS AND METHODS

### Isolation of actinomycetes from the sweetpotato

Three-month-old sweetpotato seedlings (*I. batatas* cv. Xuzishu8) were collected from the Xuzhou Academy of Agricultural Sciences (Xuzhou, China). Uniform, disease-free plants were transplanted at Jiangsu Normal University (Xuzhou, China). Roots were surface-sterilized with 5% (wt/vol, unless otherwise indicated, all percentage concentrations are expressed as weight/volume) sodium hypochlorite for 2 min, followed by 70% ethanol for 2 min, rinsed three times with sterile distilled water, chopped, and ground. Approximately 2 g of tissue was spread onto Gauze’s Synthetic Medium No. 1 plates and incubated at 37°C for 48 h. Colonies were purified on LB agar and maintained for further analysis (22).

### *In vitro* assays of plant-beneficial traits

Plant-beneficial traits of MEPSL1 were assayed under axenic conditions, including phosphate solubilization, nitrogen fixation, siderophore production, IAA biosynthesis, ACC deaminase activity, and extracellular enzyme secretion (cellulase, protease, and amylase). Unless otherwise stated, assays were performed in triplicate at 28°C with shaking at 150 rpm in Gause’s medium (23).

#### Phosphate solubilization

Phosphate solubilization was tested on modified Pikovskaya medium. MEPSL1 was inoculated and incubated at 28°C for 7 days. The presence of clear halos indicated solubilization (24).

#### Biological nitrogen fixation

Nitrogen-fixing ability was assessed on Ashby’s nitrogen-free medium. Growth after three successive transfers was taken as evidence of nitrogen fixation (25).

#### Siderophore production

Siderophore secretion was evaluated on chrome azurol S agar (pH 7.2). Yellow halos surrounding colonies were scored as positive (26).

#### IAA production

IAA production was determined by the Salkowski colorimetric method. Cultures were grown in Gause’s medium supplemented with 0.5 g L^−1^ L-tryptophan, incubated at 28°C with shaking (150 rpm), and centrifuged. Supernatants were filtered, mixed with Salkowski reagent, and incubated at 25°C for 30 min. A pink coloration indicated IAA production (27).

#### ACC deaminase activity

ACC deaminase activity was assayed on Dworkin & Foster (DF) minimal salt medium supplemented with 3 mM ACC as the sole nitrogen source. Growth after 7 days at 28°C was considered positive (28).

#### Extracellular protease production

Protease activity was tested on skim milk agar (SMA) containing yeast extract (5 g L^−1^), glucose (1 g L^−1^), pancreatic casein digest (5 g L^−1^), skim milk (28 g L^−1^), and agar (15 g L^−1^). Colonies producing clear halos after 7 days at 28°C were scored as protease producers (29).

#### Extracellular cellulase production

Cellulase activity was assessed on M9 minimal salts medium containing 10 g L^−1^ carboxymethyl cellulose (CMC). Plates were incubated at 28°C for 7 days and stained with iodine followed by 9% NaCl. Clear zones indicated cellulase activity (30).

#### Extracellular amylase production

Amylase activity was tested on agar medium containing soluble starch (2%), peptone (0.5%), NaCl (0.5%), and agar (2%). After 24 h incubation at 37°C, plates were flooded with 0.05% iodine solution. Transparent halos indicated starch degradation (31).

### Plant growth-promotion experiments

Strain MEPSL1 was cultured in ISP2 broth for 4 days. Seven-day-old sweetpotato seedlings were irrigated with 50 mL of culture broth. Controls received sterile ISP2 medium. Each treatment included three replicates. After 7 days, fresh weights of seedlings and roots were measured, and the relative increase in fresh weight was calculated as (weight of seedlings after inoculation − initial weight of seedlings)/initial weight of seedlings.

### Molecular characterization and phylogenetic analysis

#### Extraction of genomic DNA

Genomic DNA was extracted from MEPSL1 grown in glycerol liquid medium (32) at 28°C for 5 days with shaking (150 rpm), using the FastPure Bacterial DNA Isolation Kit (Vazyme, China). DNA quality was assessed on 0.8% agarose gels and quantified using a NanoDrop spectrophotometer.

#### 16S rRNA gene sequencing and phylogeny

The 16S rRNA gene was amplified with primers 27F and 1492R under standard PCR conditions, sequenced (Sangon Biotech, China), and compared with sequences in the NCBI BLAST database. A phylogenetic tree was constructed in MEGA11 using the maximum-likelihood method with 1,000 bootstrap replicates. The sequence was deposited in GenBank under accession no. PQ533238.1 (33).

### Genome sequencing, assembly, and annotation

#### Genome sequencing and quality control

Genomic DNA was extracted using the Wizard Genomic DNA Purification Kit (Promega, China). Genomic DNA of MEPSL1 was sequenced using PacBio Sequel IIe long-read technology (HiFi or CLR chemistry, as applicable) and Illumina paired-end sequencing. Illumina reads were quality-filtered with fastp and evaluated using FastQC v0.12.1 (https://github.com/s-andrews/FastQC). Long-read data quality was assessed using pbccs (HiFi) or NanoPlot (CLR).

Hybrid genome assemblies were generated using Flye, Unicycler, or SPAdes-hybrid (depending on input data). Assemblies were polished with Pilon (Illumina reads) and, when applicable, with Racon and Medaka for long-read correction. Assembly statistics were calculated using QUAST. Genome completeness and contamination were evaluated with BUSCO (bacteria_odb10) and CheckM (lineage_wf). Contamination screening was performed using Kraken2/Bracken and GUNC. Contigs <1 kb or with insufficient coverage were removed prior to annotation.

#### Structural and functional annotation

Structural annotation was performed using Prodigal v2.6.3 for coding sequence prediction, Barrnap v0.9 for rRNA gene identification, and tRNAscan-SE v2.0 for tRNA detection. Functional annotation combined eggNOG-mapper v2.1.12 (Clusters of Orthologous Groups [COG] categories), InterProScan v5.65-97.0 with InterPro2GO mappings (protein domains and Gene Ontology [GO] terms), and KOfamScan v1.3.0 (KEGG Orthology assignments), with pathway reconstruction in KEGG Mapper. Predicted protein functions were additionally supported by BLASTp searches against the Swiss-Prot database when needed.

#### Identification of biosynthetic gene clusters, CRISPR elements, and genomic islands

Secondary metabolite biosynthetic gene clusters were predicted using antiSMASH v6. CRISPR arrays and Cas proteins were identified using CRISPRCasFinder v4.2.20 and subtyped with CRISPRCasTyper v1.5.2; only arrays with evidence level ≥3 were retained. Genomic islands were predicted using IslandViewer 4 (IslandPath-DIMOB and SIGI-HMM) and cross-checked with Alien_Hunter v1.7.

### Metabolomic profiling

Liquid culture media were freeze-dried using a lyophilizer (Christ Beta 1–8 LD, Germany). The control group consisted of uninoculated medium, whereas the treatment group contained MEPSL1. Six biological replicates were prepared for each group. For metabolite extraction, 20 mg of freeze-dried material was transferred into a 2 mL centrifuge tube together with a 6 mm grinding bead. Samples were extracted with 400 μL of methanol/water (4:1, vol/vol) containing 0.02 mg/mL L-2-chlorophenylalanine as the internal standard. Homogenization was performed using a Wonbio-96c frozen tissue grinder (Shanghai Wanbo Biotechnology Co., Ltd.) for 6 min (−10°C, 50 Hz), followed by sonication for 30 min at 5°C and 40 kHz. Samples were then incubated at −20°C for 30 min and centrifuged at 13,000 × *g* for 15 min at 4°C. The resulting supernatants were transferred to LC–MS vials for analysis. A pooled QC sample was prepared by mixing 20 μL of supernatant from each extract.

Untargeted metabolomics was performed on a Thermo UHPLC-Q Exactive HF-X system equipped with an ACQUITY HSS T3 column (100 mm × 2.1 mm, 1.8 µm; Waters, USA) at Majorbio Bio-Pharm Technology Co., Ltd. (Shanghai, China). The mobile phases consisted of Solvent A: 0.1% formic acid in water/acetonitrile (95:5, vol/vol); Solvent B: 0.1% formic acid in acetonitrile/isopropanol/water (47.5:47.5:5, vol/vol/vol). The flow rate was 0.40 mL/min, and the column temperature was maintained at 40°C. Data were acquired in both positive (ESI+) and negative (ESI−) ionization modes. The ion source parameters were as follows: spray voltage +3,500 V (ESI+) and −3,500 V (ESI−), sheath gas 50 arb, auxiliary gas 13 arb, and capillary temperature 425°C. MS/MS spectra were obtained using stepped normalized collision energy (NCE 20–40–60). Full MS resolution was set to 60,000, and MS/MS resolution to 7,500. Data were collected in data-dependent acquisition mode across an *m*/*z* range of 70–1,050.

#### Data processing, feature extraction, and metabolite annotation

Raw LC–MS data were processed using the vendor pipeline at Majorbio, incorporating peak picking, alignment, retention-time correction, and feature extraction. Metabolite features were annotated by matching MS/MS spectra and accurate masses against multiple databases, including HMDB, METLIN, MassBank, mzCloud (Thermo Scientific), KEGG Compound.

Annotation levels follow the Metabolomics Standards Initiative (MSI), with Level 2 (putatively annotated compounds based on spectral library similarity) as the primary confidence category.

#### Statistical analysis and identification of differential metabolites

Processed data matrices underwent multivariate and univariate analyses. Orthogonal partial least squares discriminant analysis was used to calculate variable importance in projection (VIP) scores, and Student’s *t*-tests were applied for univariate comparison.

Metabolites were considered differentially accumulated metabolites when meeting both criteria: VIP > 1.0 and *P* < 0.05. These metabolites represent features whose abundance differs significantly between the MEPSL1-treated and control media.

### Quantitative real-time PCR

Leaves from sweetpotato seedlings in both the treatment and control groups were collected for RNA extraction. Total RNA was isolated using the RNA Pure Plant Kit (Vazyme Biotech Co., Ltd., Nanjing, China) following the manufacturer’s instructions. First-strand cDNA was synthesized using the NaviScript RT Master Mix with gDNA Sweeper (Synomebio Co., Ltd., Shanghai, China). Gene-specific primers targeting key enzymes in the vitamin E biosynthetic pathway were designed based on our previously generated transcriptome data set from sweetpotato leaves and are listed in Table S1.

Quantitative real-time PCR (qRT-PCR) was performed using the NaviScript Pro SYBR Green qPCR Master Mix-Blue (Synomebio Co., Ltd., Shanghai, China) on a StepOne Plus Real-Time PCR System (ABI, Los Angeles, CA, USA). ACTIN was used as the internal reference gene. Relative transcript levels were calculated using the 2^−ΔΔCt^ method, in which ΔCt values of the treatment groups were normalized to those of the corresponding control group. All qRT-PCR data points were retained for analysis, and outliers were excluded if CT variance exceeded 1.5 (34). The number of biological and technical replicates for each group is indicated in the legend for Fig. 6B.

The thermal cycling program was as follows: 95°C for 30 s, followed by 45 cycles of 95°C for 10 s and 60°C for 30 s. All qRT-PCR assays were performed using at least three biological replicates (unless stated otherwise).

### Tocopherol quantification

Sweetpotato seedlings were irrigated with MEPSL1 culture or sterile water (control) and incubated at 28°C for 5 days. Leaves were harvested, and α-tocopherol and γ-tocopherol were quantified using a commercial Vitamin E HPLC Assay Kit (Solarbio Life Sciences, Beijing, China; Cat. BC4894) according to the manufacturer’s protocol. The kit provides purified α-tocopherol and γ-tocopherol standards and required reagents for calibration and sample preparation.

Prior to injection, samples were filtered through 0.22 µm membrane filters. Chromatographic analysis was performed on a Kromasil C18 column (250 mm × 4.6 mm, 5 µm). An isocratic elution was used with 100% HPLC-grade methanol as the mobile phase at a flow rate of 0.80 mL min⁻¹ and a column temperature of 35°C. Detection was carried out at 295 nm using a diode-array detector. Quantification was based on five-point calibration curves for α-tocopherol and γ-tocopherol (*R*² ≥ 0.998).

### Statistical analysis

All two-group comparisons were assessed using two-tailed unpaired Student’s *t*-tests, with statistical significance defined as *P* ≤ 0.05. Data are presented as mean ± SD, based on at least three biological replicates per group for all experiments.

## RESULTS

### Isolation and identification of a growth-promoting endophyte

Endophytic isolates were obtained from sweetpotato using different enrichment media. To evaluate growth-promoting activity, single colonies were cultured in liquid medium and applied to seedlings. Among the colonies tested, one strain from ISP2 medium (designated MEPSL1) significantly enhanced seedling growth compared with the sterile control. After 7 days, seedlings treated with MEPSL1 showed a significantly increased biomass accumulation.

**Fig 1:**
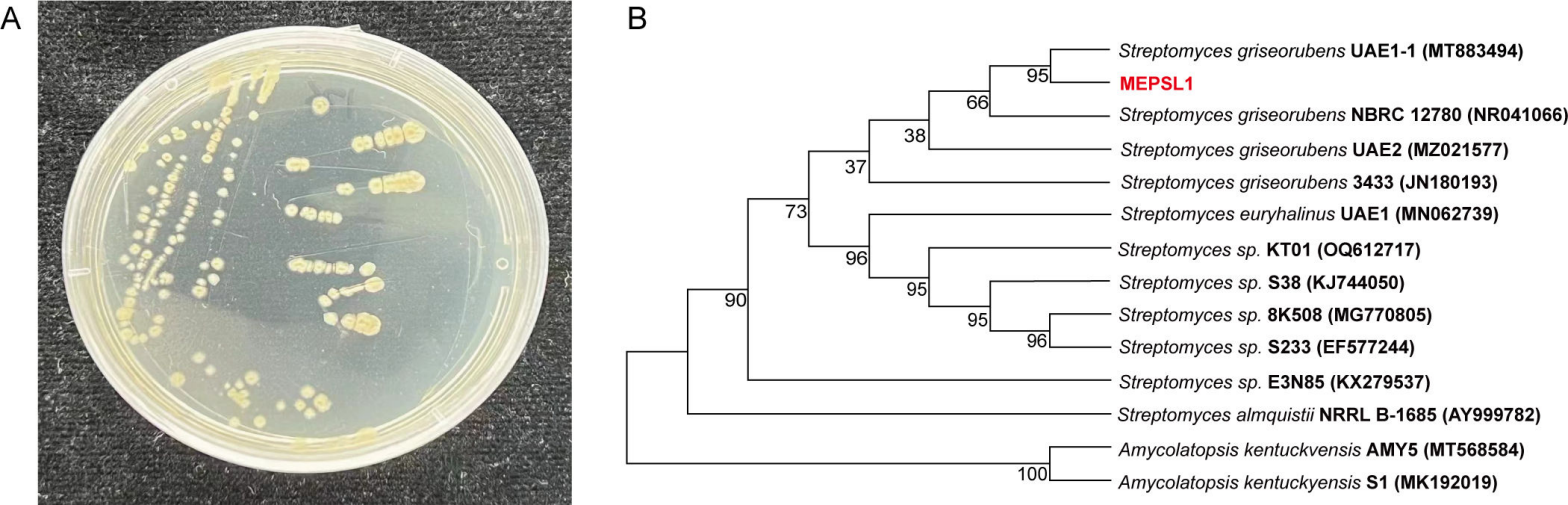
Morphological characteristics and phylogenetic analysis of MEPSL1. (**A**) Colony morphology of strain MEPSL1 grown on ISP2 medium. (**B**) Maximum-likelihood phylogenetic tree based on 16S rRNA gene sequences showing the relationship of strain MEPSL1 to closely related taxa. The tree was constructed using MEGA 11, and bootstrap support values (>50%) are indicated at the nodes.

The isolate formed colonies with a compact surface, white aerial mycelia, and pale-yellow spores, features typical of the genus *Streptomyces* (Fig. 1A). Both substrate and aerial mycelia grew vigorously without fragmentation. PCR amplification of the 16S rRNA gene (1,459 bp) using primers 27F/1492R followed by EzTaxon analysis showed high similarity to *S. griseorubens*. Phylogenetic analysis placed the strain in the *S. griseorubens* clade (Fig. 1B). On the basis of these observations, the isolate was identified as *S. griseorubens* MEPSL1.

### Growth-promoting traits of MEPSL1

**Fig 2:**
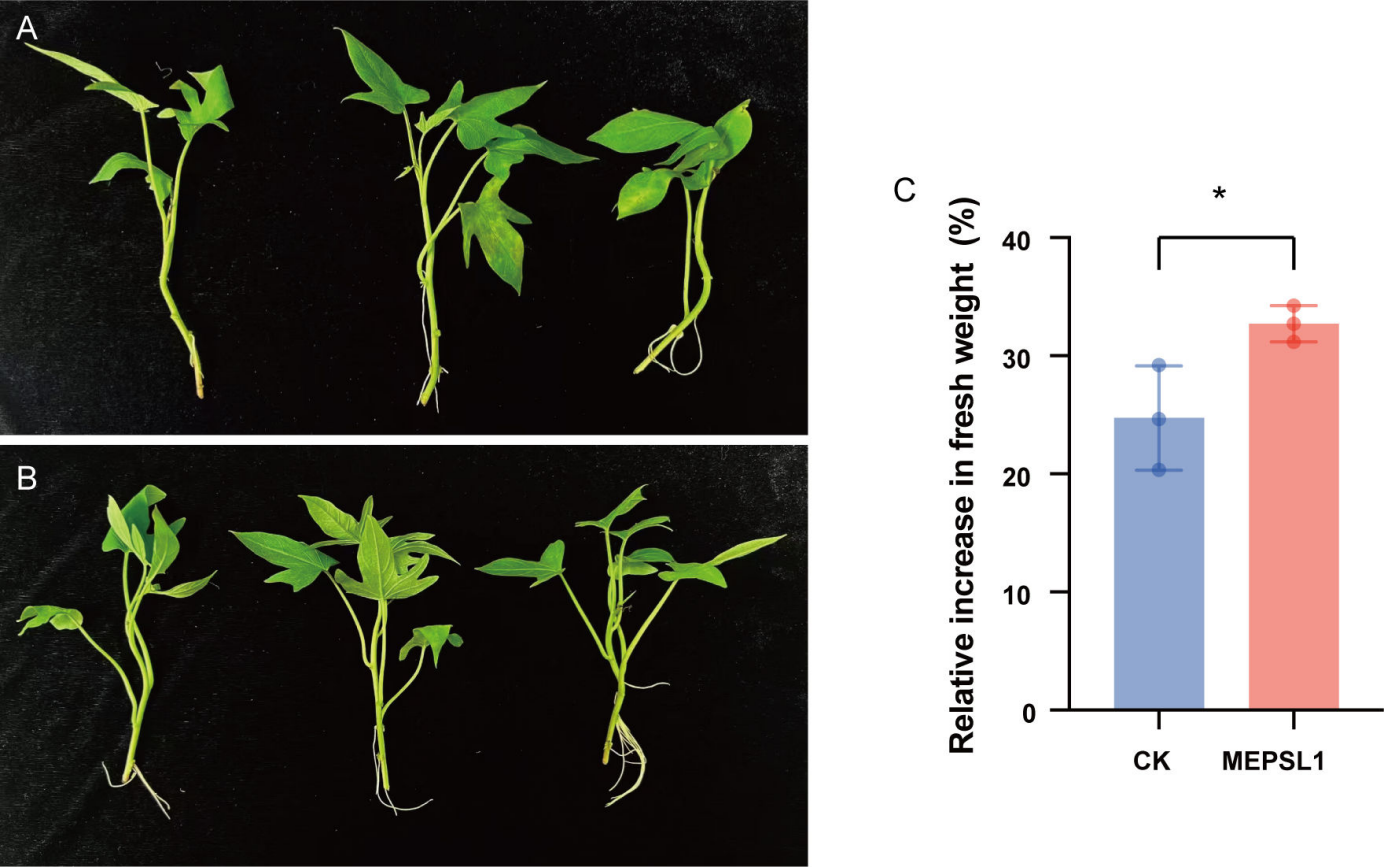
Effect of MEPSL1 application on sweetpotato seedling growth. Phenotypic comparison of control (CK) (**A**) and MEPSL1-treated (**B**) seedlings after 7 days. (**C**) Relative increase in fresh weight of sweetpotato seedlings after 7 days. Data are presented as mean ± SD (*n* = 3). Statistical significance was determined by Student’s *t*-test; *P ≤ 0.05.

To examine the plant growth-promoting potential of strain MEPSL1, we evaluated its effects on sweetpotato seedling growth. Seven days post-inoculation, sweetpotato seedlings treated with MEPSL1 demonstrated distinct growth parameters (Fig. 2A and B). In comparison to the non-inoculated control group, the growth rate of sweetpotato seedlings inoculated with strain MEPSL1 showed a significant increase (Fig. 2C).

**Fig 3:**
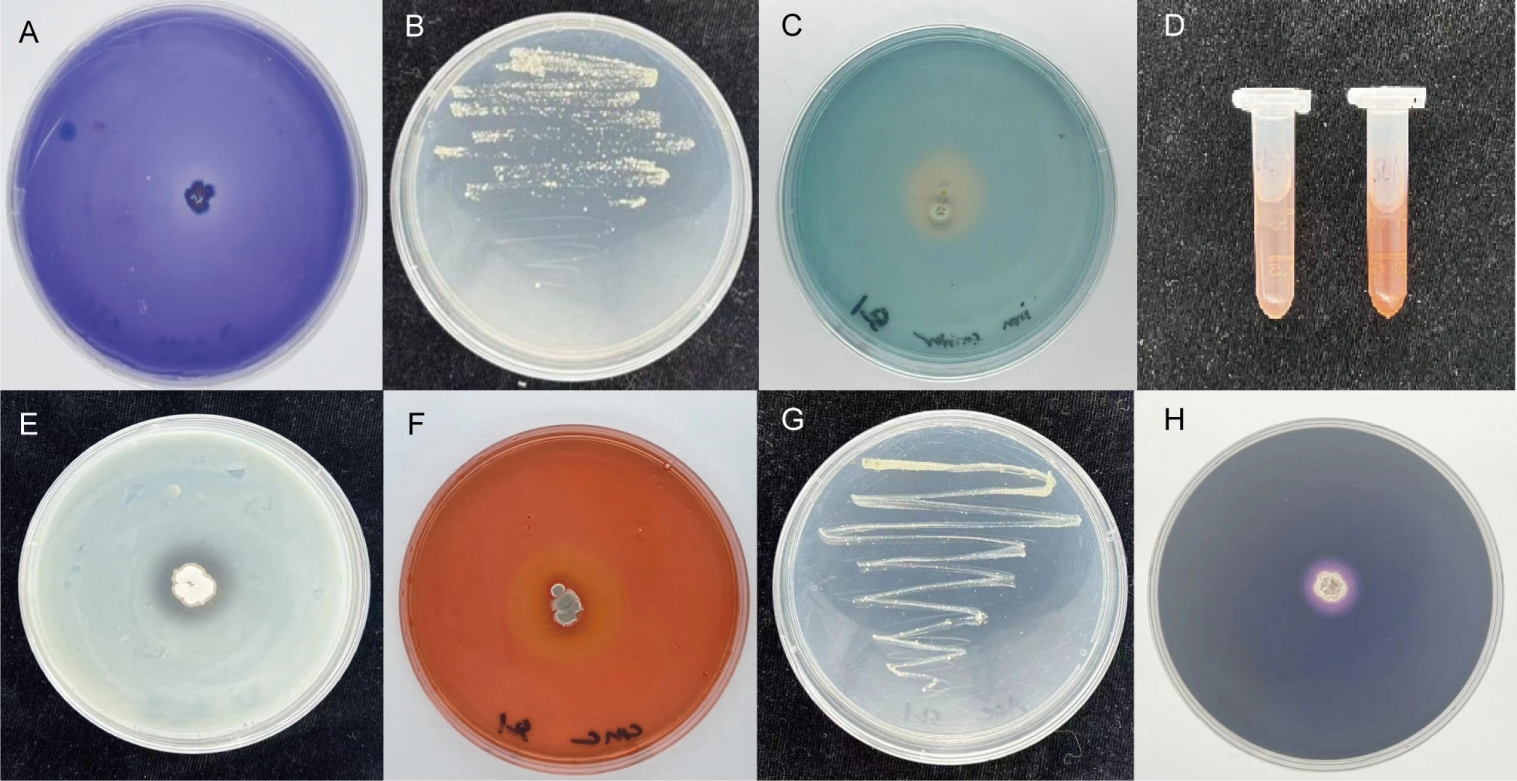
Functional validation of plant growth-promoting traits and extracellular enzyme activities of MEPSL1. Phosphate solubilization (PS) activity (**A**), nitrogen fixation activity (**B**), siderophore production (**C**), IAA production (**D**), protease activity (**E**), CMC degradation (**F**), ACC deaminase activity (**G**), and amylase activity (**H**) were evaluated to assess the plant growth-promoting potential of MEPSL1.

The functional potential of MEPSL1 was examined *in vitro*. On Pikovskaya medium, the strain produced clear halos after 7 days, confirming phosphate solubilization (Fig. 3A). Successive transfers on Ashby’s nitrogen-free medium indicated nitrogen-fixing activity (Fig. 3B). Siderophore production was evident from yellow halos on chrome azurol S agar (Fig. 3C). MEPSL1 also produced IAA (Fig. 3D).

Extracellular enzyme assays further showed activity for protease (clear halos on SMA medium, Fig. 3E), cellulase (transparent zones on CMC medium, Fig. 3F), amylase, and ACC deaminase (Fig. 3G and H). These traits collectively suggest that MEPSL1 can improve nutrient mobilization and support root development, consistent with the observed increase in seedling biomass.

These results align with previous reports on plant-beneficial streptomycetes. For example, *Streptomyces pactum* Act12 improved mustard biomass and chlorophyll content, and *Streptomyces griseoincarnatus* RB7AG enhanced rice growth under salt stress (35, 36). Such findings place MEPSL1 within a broader group of endophytic streptomycetes that combine growth promotion with stress tolerance benefits.

### Genome features of MEPSL1

The genome of MEPSL1 was sequenced to investigate the genetic basis of its plant-beneficial traits. The genome is 7,533,564 bp in size, with a GC content of 71.82%, and encodes 8,874 coding sequences, 72 tRNAs, 2 rRNAs, and 52 sRNAs. Twenty-four biosynthetic gene clusters associated with secondary metabolism were detected. The complete genome is deposited in GenBank (PRJNA1128792) (Table 1).

**TABLE 1:** General genome features of strain MEPSL1

Feature	Number or contents
Genome size (bp)	7,533,564
G + C content (%)	71.82%
Coding gene number	8,874
tRNA	72
rRNA operons	2
16S rRNA	1
23S rRNA	1
5S rRNA	0
sRNA	52
Secondary metabolite gene clusters	24
CRISPRs	26
Genomic island	4
Accession number	PRJNA1128792

**Fig 4:**
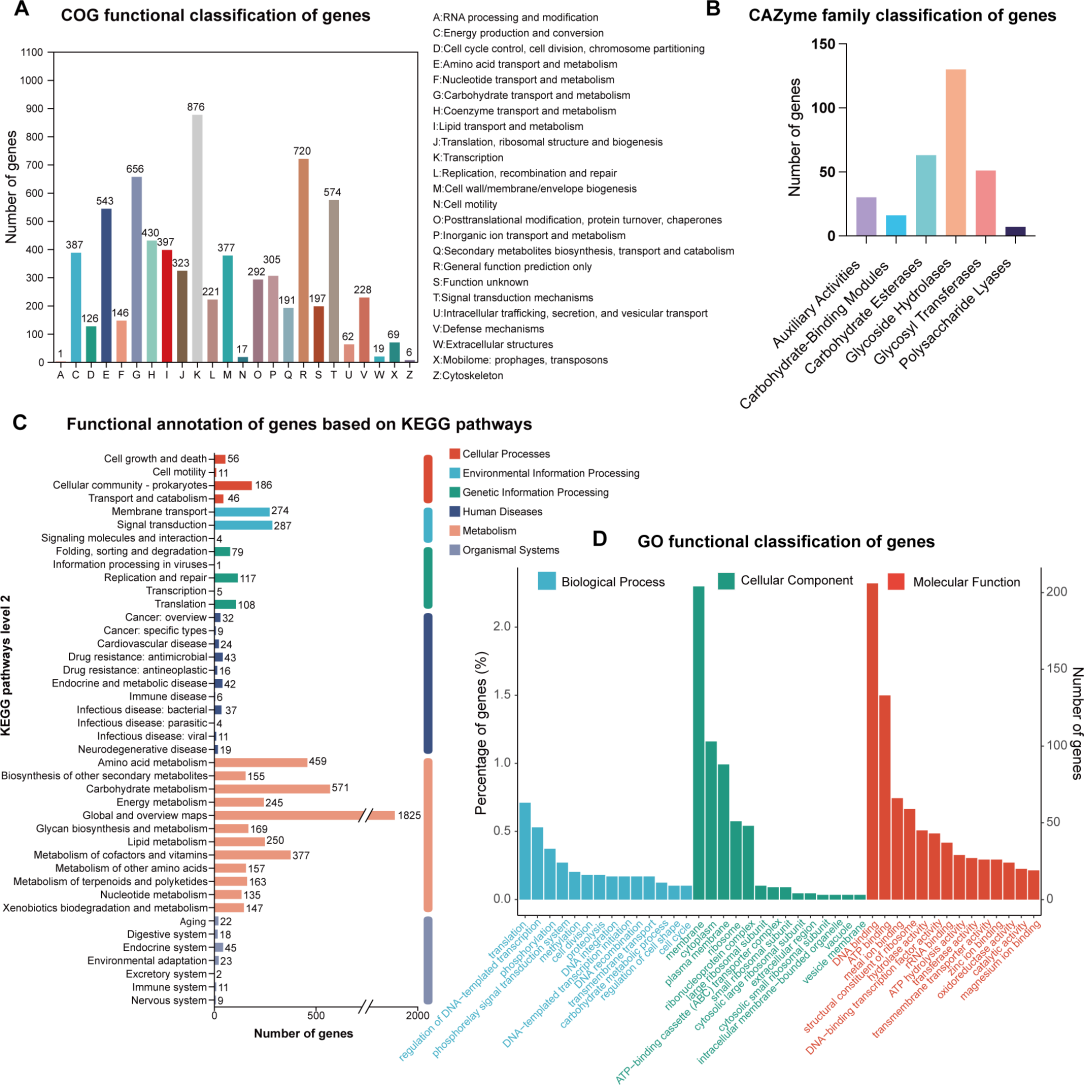
Functional annotation and classification of genes in MEPSL1. (**A**) Functional classification of genes based on the COG database. (**B**) Distribution of genes among carbohydrate-active enzyme (CAZyme) families. (**C**) Functional annotation of genes based on Kyoto Encyclopedia of Genes and Genomes (KEGG) pathways. (**D**) GO functional classification of genes, including the biological process, cellular component, and molecular function categories.

Annotation revealed genes related to IAA synthesis, siderophore production, nitrogen fixation, and phosphate solubilization (Table 2), consistent with functional assays. COG classification showed 6,323 annotated genes, with transcription (876), general function prediction (720), carbohydrate metabolism (656), and signal transduction (574) being dominant categories (Fig. 4A). CAZy analysis identified 297 carbohydrate-active enzymes, dominated by glycoside hydrolases (130) and esterases (63) (Fig. 4B).

**TABLE 2:** Genes related to plant growth

PGP traits	Genes
IAA	trpA, trpB, trpC, trpD, trpE, trpS
Siderophore	fhuB, fhuC, fhuD, fepC, fepD, fepG, ftsK, ftsW, ftsQ, ftsZ, ftsH, ftsX, ftsY
Nitrogen generation	glnA, glnB, glnD, glnE, nasT, nasC, nasB, gltA, gltB, gltC, gltD, gltX
Phosphate solubilization and uptake	pstA, pstB, pstC, pstS, phnO, phnB, phnW, phnA, phoH, phoD, phoR, phoP, phoU, phoB, phoA

KEGG mapping revealed 5,338 genes assigned to the four major KEGG functional categories relevant to microbial biology—Metabolism, Genetic Information Processing, Environmental Information Processing, and Cellular Processes—with 4,653 genes falling under Metabolism, the largest category (Fig. 4C). GO analysis indicated that DNA binding, ATP binding, and metal binding were the most common molecular functions. Genes involved in translation and regulation of DNA-templated transcription were enriched under biological processes, while cellular component annotation highlighted membrane-associated and cytoplasm-associated genes (Fig. 4D). These features suggest strong metabolic adaptability, which likely supports the strain’s endophytic lifestyle.

### Metabolomic profiling of MEPSL1

**Fig 5:**
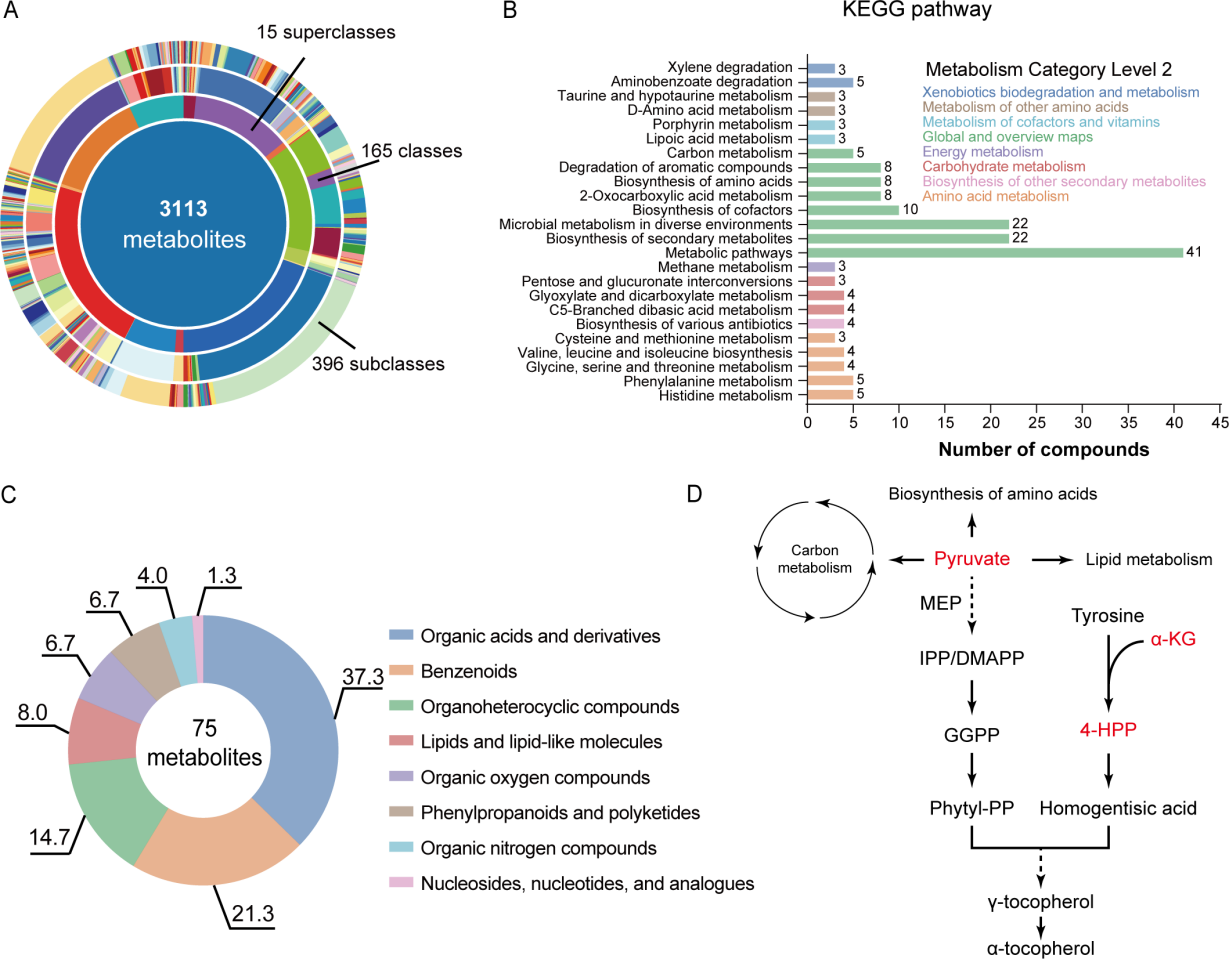
Metabolomic profiling of MEPSL1 culture supernatant and its potential links to γ-tocopherol biosynthesis. (**A**) Overview of metabolites detected by untargeted metabolomic analysis in both sterile medium (CK) and MEPSL1 culture supernatant, including metabolites annotated at confidence level ≤2. Metabolites are categorized according to their superclass, class, and subclass annotations. (**B**) Unique and increased metabolites identified in MEPSL1 relative to sterile medium, classified by KEGG pathways. Pathways are shown at KEGG Level 3, with colors indicating their corresponding KEGG Level 2 metabolic categories. (**C**) Donut plot summarizing the compound class distribution of significantly increased metabolites in MEPSL1 culture supernatant (log_2_ fold change ≥ 0.8), with percentages indicating the relative contribution of each compound class. (**D**) Schematic representation highlighting key MEPSL1-enriched metabolites—including pyruvate, α-ketoglutarate, and 4-hydroxyphenylpyruvate (4-HPP)—that are biochemically connected to the γ-tocopherol biosynthetic pathway in plants. Solid arrows indicate established plant metabolic steps, whereas dashed arrows represent proposed or indirect contributions inferred from metabolomic enrichment rather than direct metabolic tracing.

Untargeted metabolomic analysis of MEPSL1 culture supernatants and sterile medium (CK) detected a total of 3,113 metabolites annotated at MSI Levels 1–2. These metabolites were classified into 15 superclasses, 165 classes, and 396 subclasses, indicating substantial chemical diversity across both conditions (Fig. 5A). Comparison between MEPSL1 cultures and sterile medium identified metabolites uniquely detected in or increased in abundance in the MEPSL1 supernatant. These metabolites were assigned to KEGG pathways at Level 3, with pathway categories grouped by their corresponding KEGG Level 2 functional classes (Fig. 5B). At the Level 2 scale, these pathways were mainly associated with carbon metabolism, biosynthesis of amino acids, lipid metabolism, and metabolism of cofactors and vitamins.

Metabolites showing increased abundance in MEPSL1 cultures (log_2_ fold change [FC] ≥ 0.8) were further analyzed for chemical composition. Classification by compound class showed that these metabolites were primarily distributed among carboxylic acids and derivatives (25.3%), benzenoids (13.3%), keto acids and derivatives (6.7%), and organic oxygen compounds (6.7%), as summarized in donut plots representing their relative proportions (Fig. 5C).

Among the enriched metabolites, pyruvate (log_2_FC = 4.5), α-ketoglutarate (log_2_FC = 2.5), and 4-HPP (log_2_FC = 5.3) were identified as compounds biochemically connected to the γ-tocopherol biosynthetic pathway in plants (Fig. 5D). Pyruvate contributes to plastidial isoprenoid biosynthesis via the 2-C-methyl-D-erythritol 4-phosphate (MEP) pathway, 4-HPP serves as the immediate precursor of homogentisate, and α-ketoglutarate may support aminotransferase-dependent reactions in aromatic amino acid metabolism. Together, these results define a metabolic context linking MEPSL1-derived metabolites to pathways relevant to γ-tocopherol biosynthesis.

### Effect of MEPSL1 on tocopherol biosynthesis in sweetpotato

**Fig 6:**
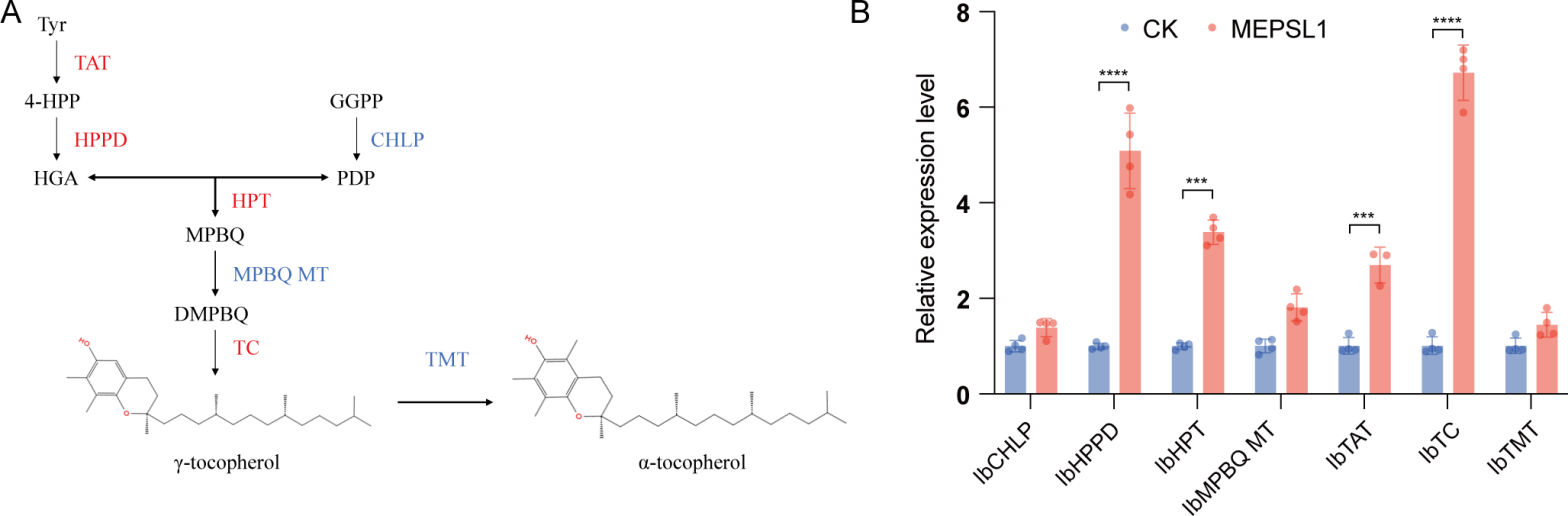
Expression analysis of tocopherol biosynthesis-related genes in sweetpotato. (**A**) Schematic representation of the tocopherol biosynthetic pathway. (**B**) Relative expression levels of tocopherol biosynthesis-related genes in sweetpotato seedlings treated with MEPSL1 and in control (CK) plants, determined by quantitative real-time PCR (qRT-PCR). Relative transcript levels were calculated using the 2^⁻ΔΔCt^ method, with normalization to the internal reference gene and to the corresponding CK samples. Data are presented as mean ± SD; *n* = 4 for all groups except IbTAT (*n* = 3). Statistical significance was assessed using Student’s *t*-test (*P ≤ 0.05, **P ≤ 0.01, ***P ≤ 0.001, and ****P ≤ 0.0001). Tyr, tyrosine; α-KG, α-ketoglutaric acid; TAT, tyrosine aminotransferase; 4-HPP, 4-hydroxyphenylpyruvate; HPPD, 4-hydroxyphenylpyruvate dioxygenase; HGA, homogentisate; GGPP, geranylgeranyl diphosphate; CHLP, geranylgeranyl reductase; PDP, phytyl diphosphate; HPT, homogentisate phytyltransferase; MPBQ, methylphytylbenzoquinol; MBPQ MT, MPBQ methyltransferase; DMPBQ, dimethylphytylbenzoquinol; TC, tocopherol cyclase; and TMT, tocopherol methyltransferase.

Given the metabolomic evidence, we examined whether MEPSL1 influences vitamin E biosynthesis *in planta*. Based on published sweetpotato genome data, key genes in the tocopherol pathway were selected for qRT-PCR analysis. Inoculated plants showed significant upregulation of IbHPPD, IbHPT, IbTAT, and IbTC (Fig. 6).

**Fig 7:**
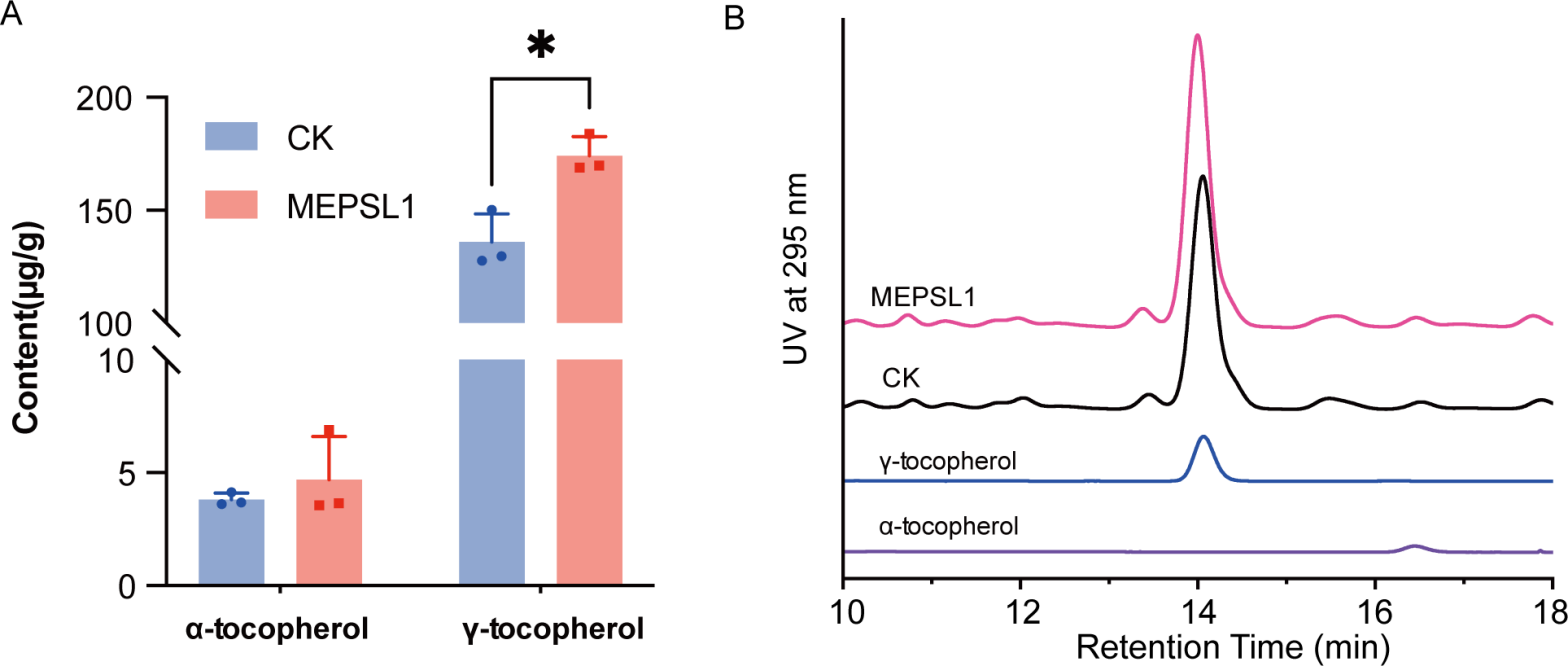
MEPSL1 increases tocopherol accumulation in sweetpotato leaves. (**A**) Contents of α-tocopherol and γ-tocopherol in sweetpotato leaves from control (CK) and MEPSL1-treated plants, as determined by HPLC. Data are presented as mean ± SD (*n* = 3). Statistical significance was assessed using Student’s *t*-test (*P ≤ 0.05). (**B**) Representative (*n* = 3) HPLC chromatograms recorded at 295 nm showing α-tocopherol and γ-tocopherol peaks in CK and MEPSL1-treated samples, with corresponding standards indicated.

As shown in Fig. 7A and B, HPLC confirmed increases in both α-tocopherol and γ-tocopherol. Levels of α-tocopherol rose from 135.87 to 174.18 μg/g DW, and γ-tocopherol increased from 3.81 to 4.70 μg/g DW. Although the changes are moderate, they provide biochemical evidence that MEPSL1 affects host secondary metabolism.

The results suggest involvement of both microbial metabolite supply and transcriptional regulation of host pathways. Endophytes in the genus Epichloë, for example, enhance tocopherol levels by modulating host nitrogen metabolism, while fungal strains such as *Colletotrichum tofieldiae* activate host genes involved in antioxidant biosynthesis. MEPSL1 may act in a similar dual fashion: by producing precursors (α-ketoglutarate, 4-HPP, pyruvate) and by inducing host gene expression.

These findings connect to earlier demonstrations that HPPD and HPT are essential regulators of tocopherol biosynthesis. Targeted mutations in HPT reduce tocopherol levels dramatically, and overexpression of IbHPPD and IbHPT in sweetpotato enhances vitamin E accumulation. The induction of these genes by MEPSL1, therefore, provides a plausible explanation for the observed increases in tocopherol content.

## DISCUSSION

*Streptomyces* species are widely recognized as plant-associated microbes with roles in nutrient mobilization, hormone modulation, and biocontrol (37). Consistent with this, MEPSL1 exhibited multiple plant growth-promoting traits, including IAA biosynthesis, ACC deaminase activity, phosphate solubilization, and nitrogen fixation. Genome sequencing further supported the presence of genes responsible for these functions. Such traits have been documented in other beneficial streptomycetes and are consistent with their ability to enhance seedling vigor and stress resilience (38).

In addition to promoting growth, inoculation of MEPSL1 further increased γ-tocopherol levels in leaves. qRT-PCR results showed upregulation of HPPD, HPT, TC, and related tocopherol pathway genes, suggesting that MEPSL1 might modulate host antioxidant metabolism. Similar effects have been reported for other plant-associated microbes, which can influence host secondary metabolism by altering nutrient status, redox homeostasis, or hormone signaling networks (39).

In addition, metabolomic profiling revealed that MEPSL1 cultures produce several metabolites—such as pyruvate, α-ketoglutarate, and 4-HPP—that are biochemically positioned upstream of homogentisate formation in the tocopherol pathway (40, 41). Together with the observed upregulation of tocopherol biosynthetic genes in sweetpotato, these findings suggest a potential link between MEPSL1 metabolism and host γ-tocopherol accumulation.

Previous studies indicate that microbial organic acids and aromatic intermediates can modulate plant secondary metabolism by affecting metabolic flux or redox signaling (42, 43). Based on this precedent, we propose a hypothetical working model in which MEPSL1-derived metabolites may act as precursors or metabolic cues that could prime tocopherol biosynthesis in the host. However, our current data do not demonstrate direct uptake or incorporation of microbial metabolites into plant tissues, and this remains to be further investigated.

Future studies, including stable-isotope tracing, metabolite transport assays, and analysis of MEPSL1 metabolic mutants, will be essential to determine whether and how microbial metabolites contribute to plant vitamin E biosynthesis. Additionally, our plant experiments were conducted at the seedling stage; thus, follow-up studies on mature plants and under field conditions will be essential to assess robustness and agricultural relevance.

### Conclusion

*S. griseorubens* MEPSL1 was isolated from sweetpotato and characterized as a plant growth-promoting endophyte with multiple beneficial traits, including hormone regulation, ACC deaminase activity, phosphate solubilization, nitrogen fixation, and secretion of extracellular enzymes. MEPSL1 inoculation enhanced sweetpotato seedling growth and increased γ-tocopherol accumulation. Genomic and metabolomic analyses indicate that MEPSL1 possesses both the genetic basis and metabolic capacity to support these functions.

While the mechanisms by which MEPSL1 influences tocopherol biosynthesis remain hypothetical, our findings provide a foundation for future mechanistic studies and highlight the potential of MEPSL1 as a microbial resource for crop bioaugmentation and nutritional enhancement. Further evaluation—including stable isotope tracing, molecular dissection of host–microbe interactions, and assessment in mature plants and field environments—will be required to determine its broader agricultural applicability.

## Data Availability

The whole-genome sequencing data generated in this study have been deposited in the NCBI Sequence Read Archive (SRA) under BioProject accession no. PRJNA1128792, with the associated SRA run SRR30330531. The non-targeted metabolomics data have been deposited in the OMIX database at the China National Center for Bioinformation/Beijing Institute of Genomics, Chinese Academy of Sciences (accession no. OMIX009800). Transcriptome sequencing data used for qRT-PCR primer design are available in the NCBI Sequence Read Archive under BioProject accession no. PRJNA1126169.

## SUPPLEMENTAL MATERIAL

Table S1 spectrum.03070-25-s0001.docx Gene-specific primers for the tocopherol biosynthesis-related genes in sweetpotato.

ASM does not own the copyrights to Supplemental Material that may be linked to, or accessed through, an article. The authors have granted ASM a non-exclusive, world-wide license to publish the Supplemental Material files. Please contact the corresponding author directly for reuse.
