# Supplementary material for: Endophytic Streptomyces griseorubens MEPSL1 from sweetpotato promotes plant growth and enhances γ-tocopherol accumulation
Source: Microbiol Spectr. 2026 Feb 17;14(4):e03070-25. doi: 10.1128/spectrum.03070-25 (PMC13055315; doi:10.1128/spectrum.03070-25)
Supplement: Table S1 — Gene-specific primers for the tocopherol biosynthesis-related genes in sweetpotato. [file spectrum.03070-25-s0001.docx]

**Endophytic *Streptomyces* *griseorubens* MEPSL1 from sweetpotato promotes plant growth and enhances γ-tocopherol accumulation**

Jingsheng Gu^a,1^, Yiming Wang^a,1^, Yu Sun^a^, Yuxuan Xu^a^, Yuanjiao Li^a^, Chunyu Lin^a^, Yue Ke^a^, Lei Kai^a#^

^a^The Key Laboratory of Biotechnology for Medicinal and Edible Plants of Jiangsu Province, School of Life Sciences, Jiangsu Normal University, Xuzhou, 221116, P. R. China

Running Head: *Streptomyces griseorubens* MEPSL1 promotes sweetpotato growth and enhances the accumulation of γ-tocopherol

#Corresponding author: Lei Kai, lkai@jsnu.edu.cn

^1^ These authors contributed equally to this work.

**SupplementaryTable1. Gene-specific primers for the tocopherol biosynthesis–related genes in sweet potato**

| **Genes** |  |  |  |  | **Primers** |
| --- | --- | --- | --- | --- | --- |
| F-HPT-qpcr |  |  |  |  | GCTTCTTCTCGGTTGTTATTG |
| R-HPT-qpcr |  |  |  |  | GGTAAGCCATTTGAAGTAGTG |
| F-IbHPPD-QPCR |  |  |  |  | TGAACTCAGTGGTGCTGGCTAA |
| R-IbHPPD-QPCR |  |  |  |  | CCTGGTCCTTCGTTGTGC |
| F-MPBQ MT-qpcr |  |  |  |  | GACCTCCCATTTCCCACT |
| R-MPBQ MT-qpcr |  |  |  |  | ATAACGCACGCTACTCCC |
| F-TC-qpcr |  |  |  |  | ACCCTCCTTGCATGTTCT |
| R-TC-qpcr |  |  |  |  | GGCACCGATACTGCTTTC |
| F-TMT-qpcr |  |  |  |  | TGAATCAGCCCTTTCCTA |
| R-TMT-qpcr |  |  |  |  | TGTGGCACCATGTAACTATG |
| F-TAT -qpcr |  |  |  |  | ATGGAAAACGGCGGCGGGTCGAAGA |
| R-TAT -qpcr |  |  |  |  | TCTTCGACCCGCCGCCGTTTTCCAT |
| F-CHLP-qpcr |  |  |  |  | CGTGAAGTTCTCGACGCTTAT |
| R-CHLP-qpcr |  |  |  |  | GGTGTACTGGAGGACATAGGG |
